# Supplementary figures and images for: A first assessment of the distribution and abundance of large pelagic species at Cocos Ridge seamounts (Eastern Tropical Pacific) using drifting pelagic baited remote cameras
Source: PLoS One. 2021 Nov 18;16(11):e0244343. doi: 10.1371/journal.pone.0244343 (PMC8601560; doi:10.1371/journal.pone.0244343)

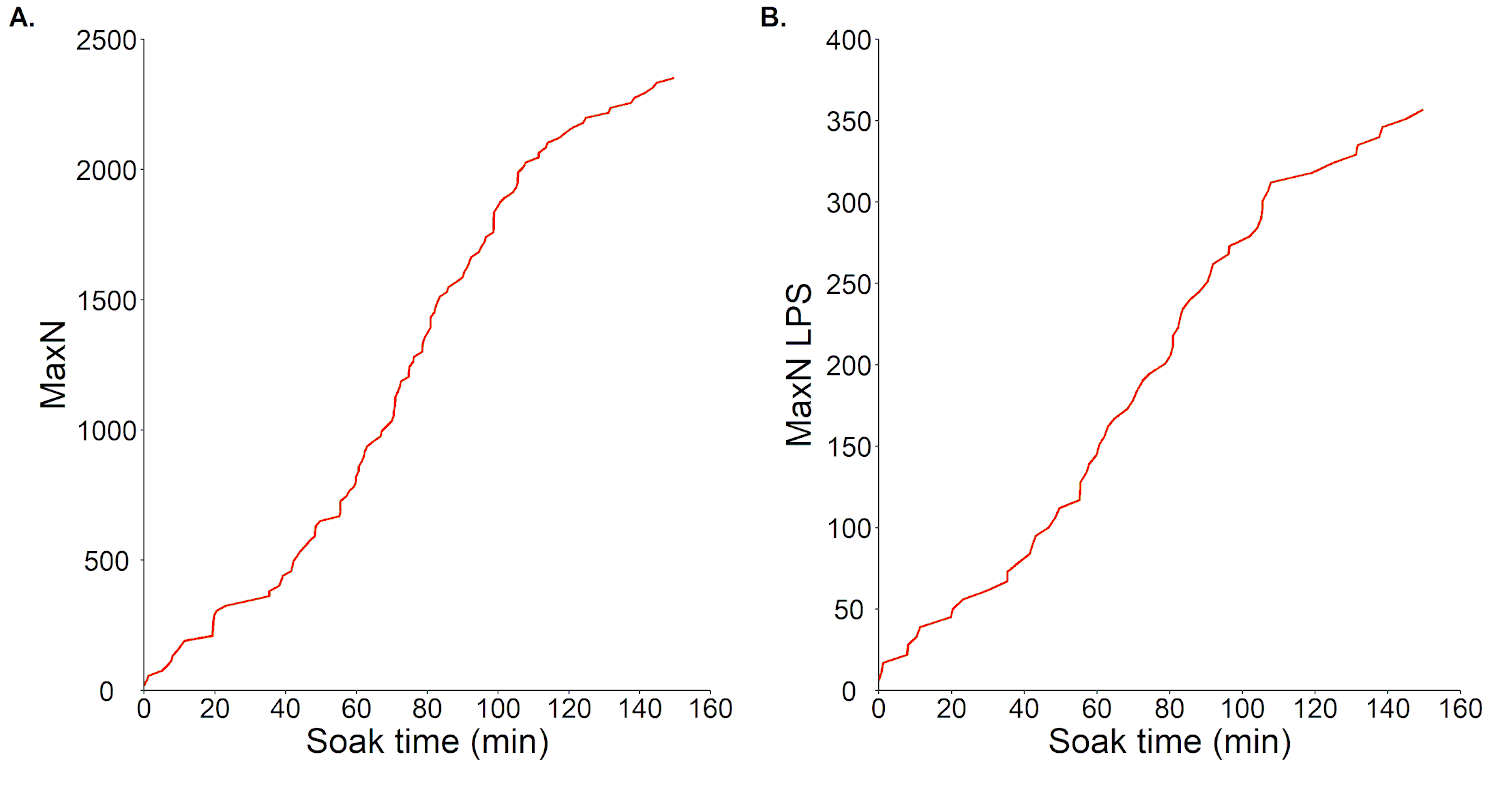

Supplement: S1 Fig — Soak time is defined as the effective recording time of Baited Remote Underwater Video Stations (BRUVS). Each deployment with 5 connected BRUVS is treated as an independent sample. (TIF) [file pone.0244343.s001.tif]

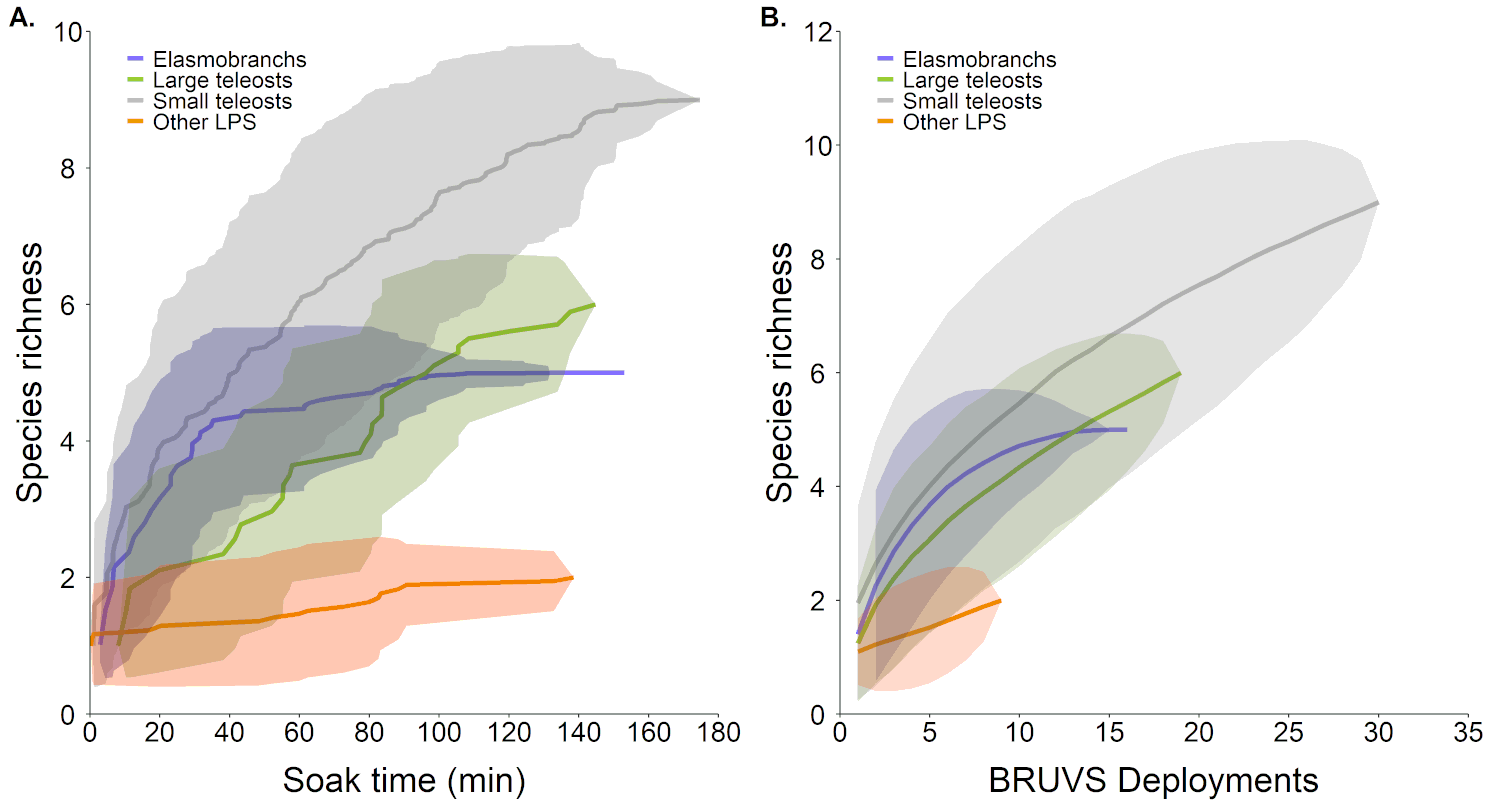

Supplement: S2 Fig — (A) Species richness of each ecological group over cumulative soak time in minutes. Soak time is defined as the effective recording time of Baited Remote Underwater Video Stations (BRUVS). (B) Species richness of each ecological group over the number of BRUVS deployments. Each deployment with five connected BRUVS is treated as an independent sample. Shade colors represent 95% confidence intervals. (TIF) [file pone.0244343.s002.tif]

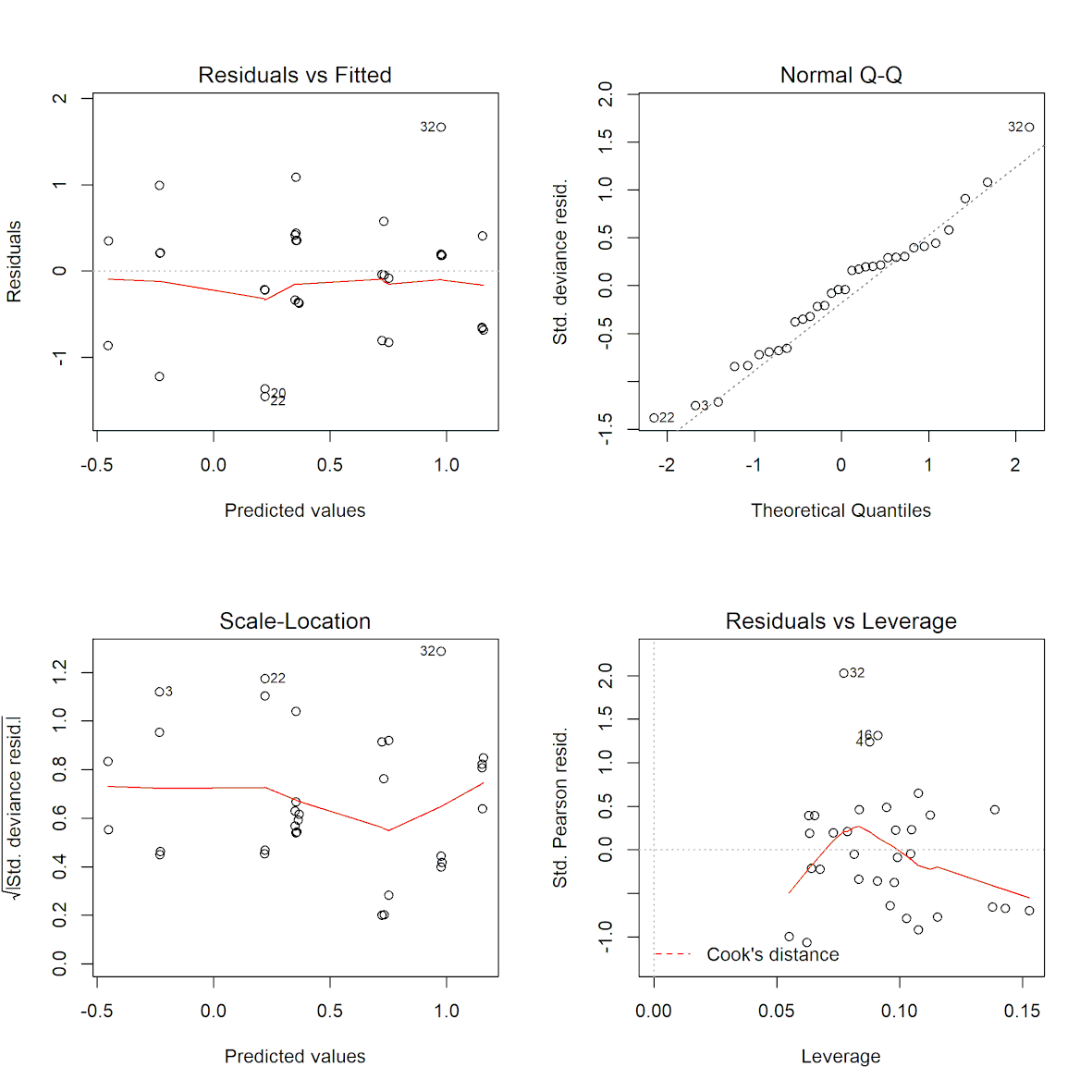

Supplement: S3 Fig — The model is presented in Table 3. (TIF) [file pone.0244343.s003.tif]

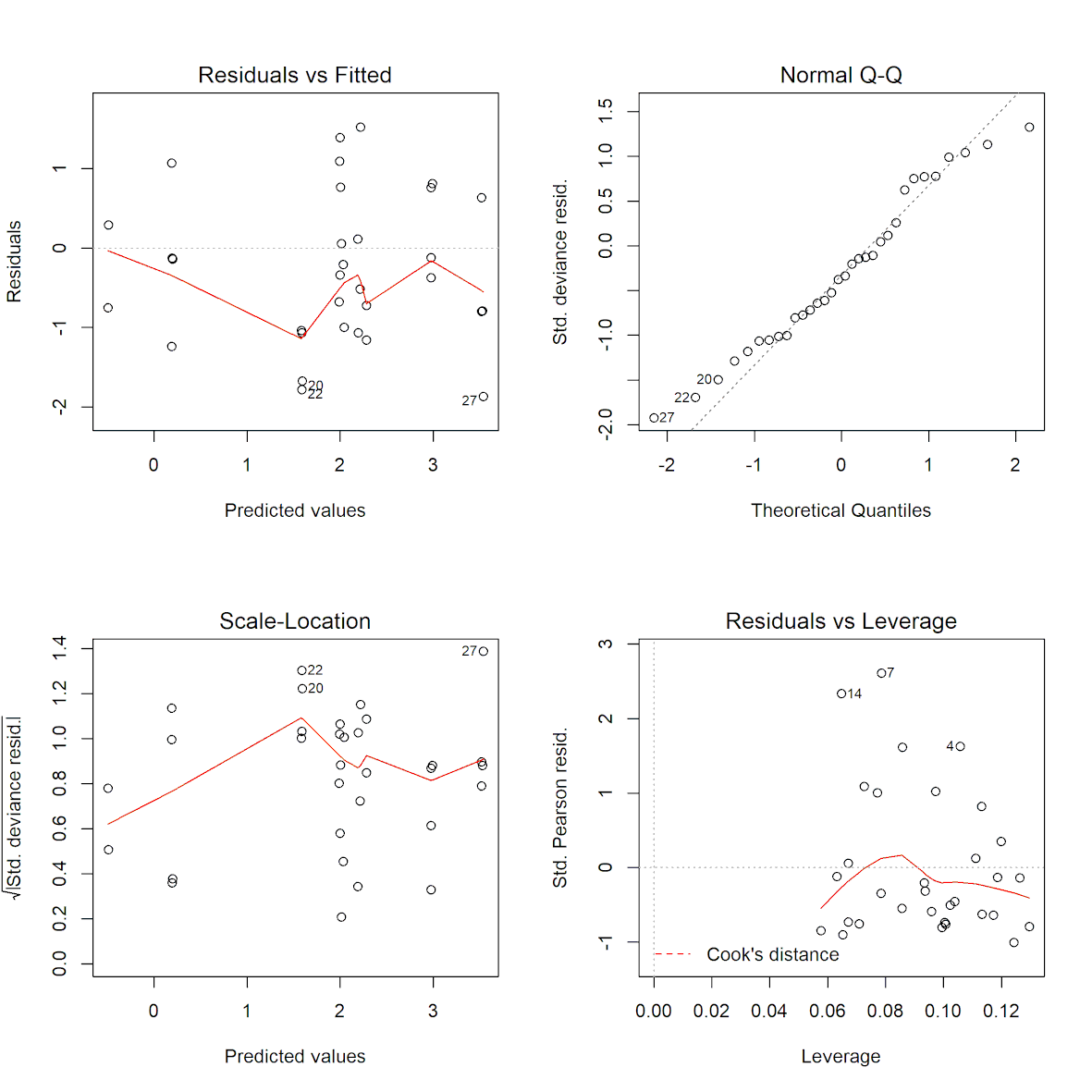

Supplement: S4 Fig — The model is presented in Table 3. (TIF) [file pone.0244343.s004.tif]

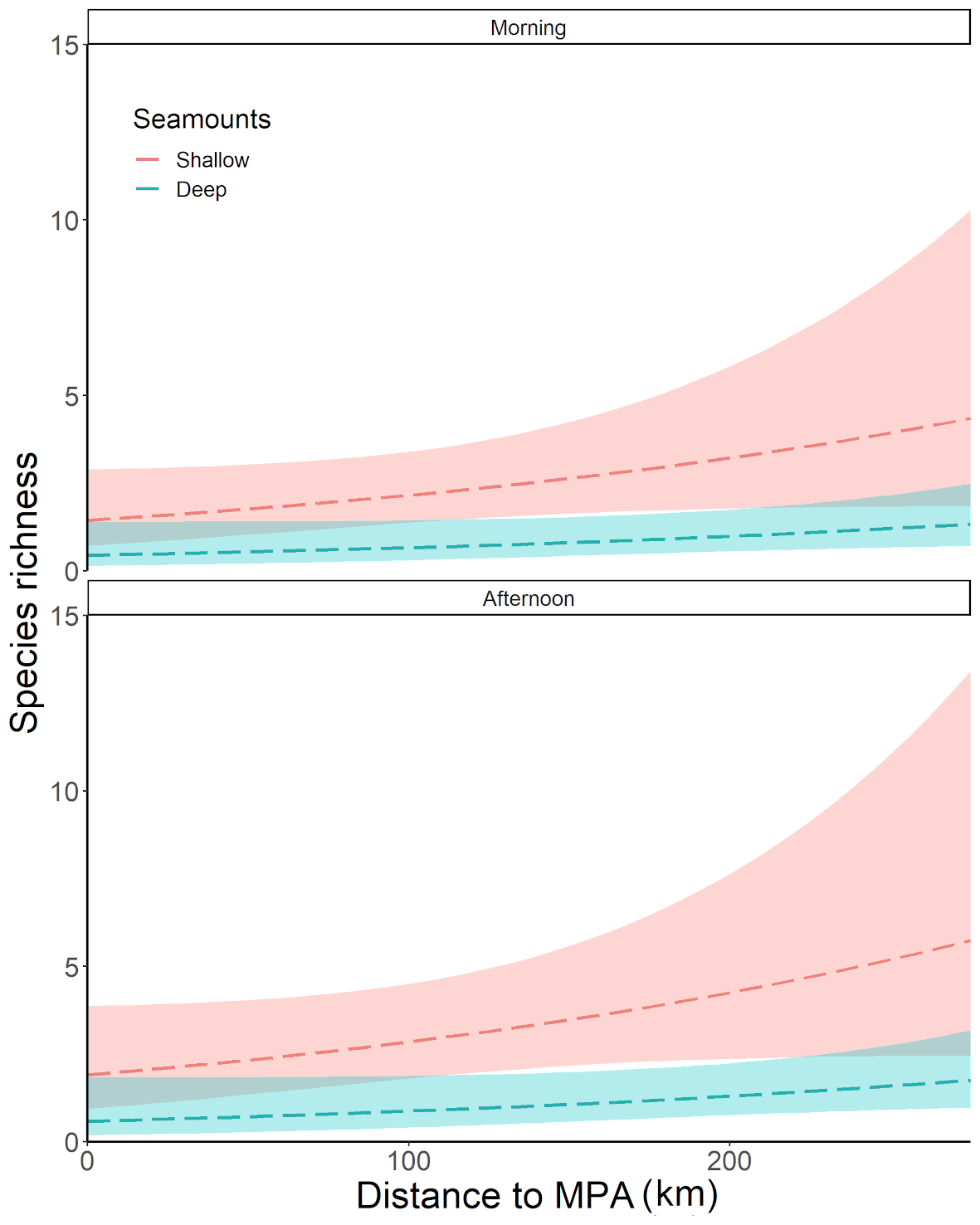

Supplement: S5 Fig — Representation of the relationship between richness of LPS and distance to nearest MPA (Cocos or Galapagos Islands) at shallow (<400 m) and deep (>400 m) seamounts during morning and afternoon deployments. (TIF) [file pone.0244343.s005.tif]

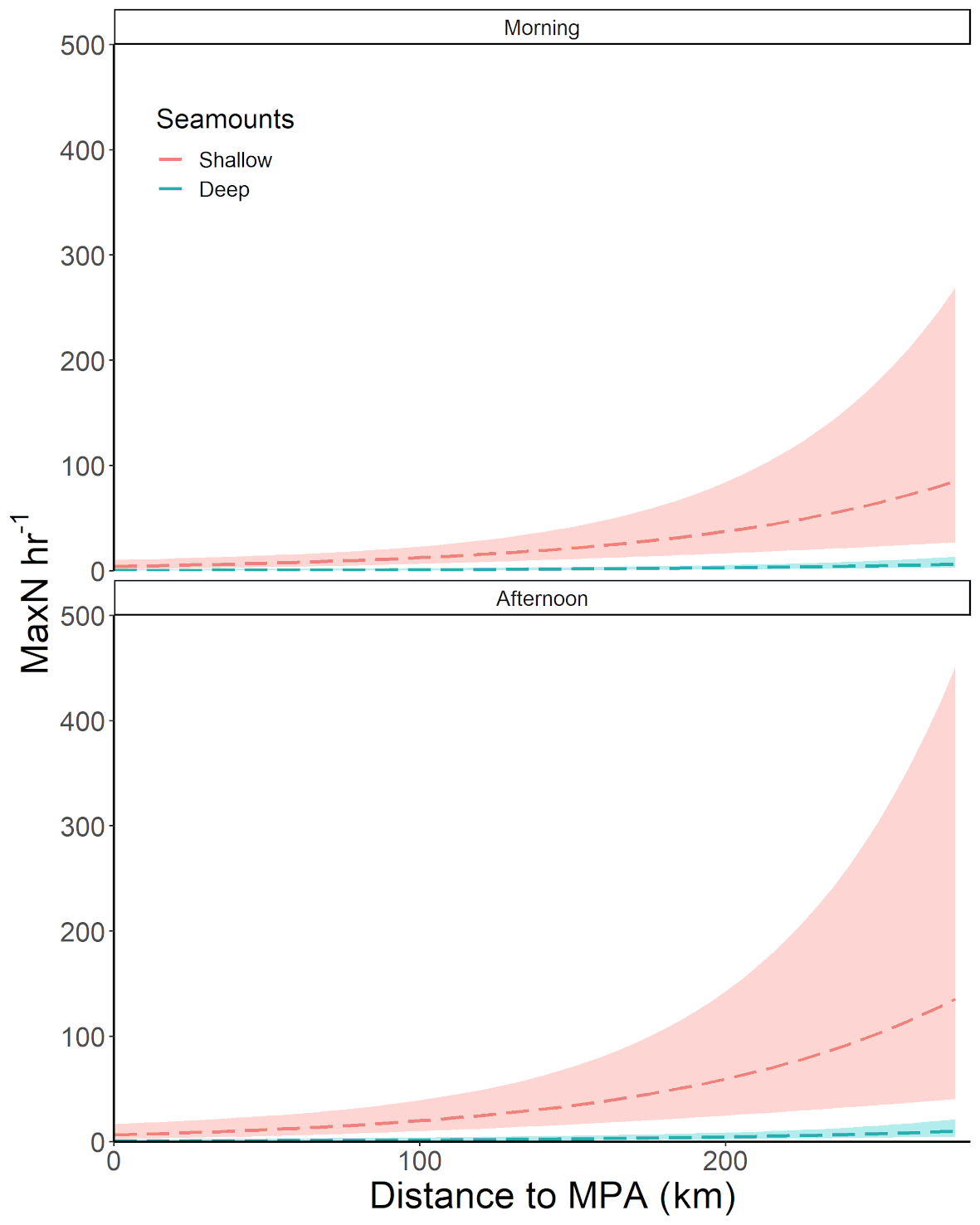

Supplement: S6 Fig — Representation of the relationship between relative abundance of LPS and distance to nearest MPA (Cocos or Galapagos Islands) at shallow (<400 m) and deep (>400 m) seamounts during morning and afternoon deployments. (TIF) [file pone.0244343.s006.tif]
