# Supplementary material for: A first assessment of the distribution and abundance of large pelagic species at Cocos Ridge seamounts (Eastern Tropical Pacific) using drifting pelagic baited remote cameras
Source: PLoS One. 2021 Nov 18;16(11):e0244343. doi: 10.1371/journal.pone.0244343 (PMC8601560; doi:10.1371/journal.pone.0244343)
Supplement: S1 Table — Relative abundance is expressed as MaxN hr-1 (maximum number of individuals of a species recorded on a single deployment standardized by soak time). MaxN hr-1 Species are abreviated as: AF) Alepisaurus ferox, AP) Alopias pelagicus, CF) Carcharhinus falciformis, CM) Chelonia mydas, CH) Coryphaena hippurus, II) Istiompax indica, IP) Istiophorus platypterus, KA) Kajikia audax, M) Mobula spp., PV) Pteroplatytrygon violacea, SL) Sphyrna lewini, TA) Thunnus albacares and TT) Trusiops truncatus. The MaxN hr-1 at a group level for the small teleost species (ST) is also presented. The sum and the mean of LPS MaxN hr-1 and species richness are also presented per seamount. Seamounts are ordered according to the sum of MaxN hr-1. Shallow seamounts (<400 m) are shown in italics. (DOCX) [file pone.0244343.s007.docx]

| **Seamounts** | **AF** | **AP** | **CF** | **CH** | **CM** | **II** | **IP** | **KA** | **M** | **PV** | **SL** | **TA** | **TT** | **ST** | **MaxN hr^-1^** | | **Richness** | |
| --- | --- | --- | --- | --- | --- | --- | --- | --- | --- | --- | --- | --- | --- | --- | --- | --- | --- | --- |
|  |  |  |  |  |  |  |  |  |  |  |  |  |  |  | **sum** | **mean±SD** | **sum** | **mean±SD** |
| *West Cocos* | 0 | 0 | 0.4 | 5.1 | 0.4 | 0.4 | 0 | 0.4 | 0 | 0 | 34.6 | 1.1 | 8.7 | 16.3 | 51.1 | 12.8 ± 6.2 | 8 | 3.8 ± 1.5 |
| *Paramount* | 0 | 0.9 | 2.3 | 0 | 0 | 0 | 0 | 0 | 0.8 | 0 | 32.5 | 0 | 0 | 29.8 | 36.4 | 9.1 ± 9.9 | 4 | 2.5 ± 1 |
| *Las Gemelas 1* | 0 | 0.4 | 0 | 18.3 | 0 | 0 | 0.4 | 0.4 | 0 | 0 | 0 | 0 | 5.9 | 15.1 | 25.4 | 6.4 ± 8.2 | 5 | 2 ± 0.8 |
| Medina 1 | 0.4 | 0 | 0 | 24.1 | 0 | 0 | 0 | 0.8 | 0 | 0 | 0 | 0 | 0 | 9.8 | 25.3 | 6.3 ± 7.5 | 3 | 1.5 ± 0.6 |
| Medina 2 | 0 | 0 | 0.4 | 15.8 | 0 | 0 | 0 | 0 | 0.5 | 0.4 | 0 | 0.4 | 0 | 17.8 | 17.5 | 4.4 ± 3.2 | 5 | 2 ± 0.8 |
| East Cocos | 0 | 0 | 0 | 0.8 | 0 | 0 | 0 | 0.4 | 0 | 0.4 | 0 | 0 | 0.8 | 32.5 | 2.4 | 0.6 ± 0.7 | 4 | 1 ± 0.8 |
| *Las Gemelas 2* | 0 | 0 | 0 | 0 | 0 | 0 | 0 | 0 | 0 | 0 | 1.5 | 0 | 0.9 | 680 | 2.4 | 1.2 ± 0.6 | 2 | 1.5 ± 0.7 |
| Medina 3 | 0 | 0 | 0 | 0 | 0 | 0 | 0 | 0.5 | 0 | 0 | 0.4 | 0 | 0 | 12.9 | 0.9 | 0.2 ± 0.3 | 2 | 0.5 ± 0.6 |
| NW Darwin | 0 | 0 | 0 | 0.5 | 0 | 0 | 0 | 0 | 0 | 0 | 0 | 0 | 0 | 21.9 | 0.5 | 0.2 ± 0.4 | 1 | 0.5 ± 0.7 |

**S1 Table.** **Relative abundance (MaxN hr**^-1^**) and richness of large pelagic species (LPS) by seamount**

Relative abundance is expressed as MaxN hr^-1^ (maximum number of individuals of a species recorded on a single deployment standardized by soak time). MaxN hr^-1^ Species are abreviated as: AF) *Alepisaurus ferox*, AP) *Alopias pelagicus*, CF) *Carcharhinus falciformis*, CM) *Chelonia mydas agassizi*, CH) *Coryphaena hippurus*, II) *Istiompax indica*, IP) *Istiophorus platypterus*, KA) *Kajikia audax*, M) *Mobula* spp., PV) *Pteroplatytrygon violacea*, SL) *Sphyrna lewini*, TA) *Thunnus albacares* and TT) *Trusiops truncatus*. The MaxN hr^-1^ at a group level for the small teleost species (ST) is also presented. The sum and the mean of LPS MaxN hr^-1^ and species richness are also presented per seamount. Seamounts are ordered according to the sum of MaxN hr^-1^. Shallow seamounts (<400 m) are shown in italics.
