# Supplementary material for: A first assessment of the distribution and abundance of large pelagic species at Cocos Ridge seamounts (Eastern Tropical Pacific) using drifting pelagic baited remote cameras
Source: PLoS One. 2021 Nov 18;16(11):e0244343. doi: 10.1371/journal.pone.0244343 (PMC8601560; doi:10.1371/journal.pone.0244343)
Supplement: Attachment — Submitted filename: Response to reviewers.docx [file pone.0244343.s012.docx]

**REVIEWER 1**

Answers to reviewer’s 1 comments. The location of the comments at the original version (lines 1) and the new version (lines 2) are presented.

| **Lines 1** | **Lines 2** | **REVIEWER’S COMMENTS** | **AUTHOR’S REPLY** |
| --- | --- | --- | --- |
| 24 | 25 | Seamounts are important features in shaping the ecology of open ocean animals. Yet, understanding fish movement patterns and how they respond to the environment is key to guide management in open ocean, not exclusively the links to seamounts which may only explain or determine in part the spatial-ecology. Suggest toning down the statement and frame the relative importance of seamounts with other variables and life history. | We agree with the reviewer. The sentence has been changed to *“Understanding the link between seamounts and large pelagic species (LPS) may provide important insights for the conservation of these species in open water ecosystems”.* |
| 28 | 29 | there are a number of telemetry studies that support the previous statement (some by the co-authors) which is in contradiction with this one. | We acknowledge that a number of telemetry studies in the region have provided important information regarding movement patterns and habitat use of LPS. However, these studies do not provide information regarding the community structure of LPS in pelagic ecosystems since they are based on the movement of one tagged individual. Since our data mainly described diversity and abundance patterns instead of describing the whole community structure we have change the sentence to: *“However, to date, research efforts to understand abundance and distribution patterns of LPS beyond the borders of these two oceanic Marine Protected Areas (MPAs) have been limited”*. We have also substitute “community structure” by abundance and/or distribution patterns at lines 66 and 75. |
| 46 | 48 | open ocean is not highly productive, seamounts are | We agree with the reviewer. The sentence has been changed to *“Overexploitation of the open ocean by industrial fisheries…”* |
| 64 | 67 | what is the definition of associated to seamount? | We agree with the reviewer that it might sound confusing without a previous definition. The sentence has been changed from “*associated to seamounts”* to *“at Cocos Ridge seamounts”.* |
| 66 | 69 | verify the citation | We verified the citation and changed (CBD, 2009) by (Secretariat of the Convention on Biological Diversity, 2009) as is presented in the report. We have also cited another study more focused on the study area (Peñaherrera et al. 2018) and changed the sentence from *“there is a greater need to survey the open ocean”* to *“there is a greater need to survey the pelagic ecosystem beyond the borders of MPAs”.* |
| 72 | 76 | not necessarily, many species may never aggregate in the open ocean | We agree with the author and changed the sentence to *“to understand how the pelagic community is distributed in the open ocean”.* |
| 129 | 135 | Check | We agree this was not the correct reference. We changed it by Prince & Goodyear 2007. Consequences of Ocean Scale Hypoxia Constrained Habitat for Tropical Pelagic Fishes |
| 131 | 137 | conclusions must consider the short term nature of the study and potential seasonal effects | We completely agree with the reviewer and believe that we have already clarified in the manuscript that the small temporal scale of this study is a limitation. For example:  Line 481: *“we need a higher spatial and temporal replication to elucidate if LPS along Cocos Ridge are positively associated to seamounts presence”…. “ Future research should consider deploying drifting-pelagic BRUVS at different distances from seamounts covering different seasons in order to elucidate the aggregation effect of these topographic features on LPS [53]”*  Line 557: “*Although our study is limited by a short temporal and spatial replication, our results can be used as a first approach to guide future studies using drifting-pelagic BRUVS in the ETP”*  Line 547: “*Future studies with higher temporal and spatial survey efforts, including seasonal variation [106] and the interannual ENSO variability [50], are necessary to better understand the environmental variations at each seamount and its effect on LPS”* |
| 141 | 150 | the information available seems limited and but not likely significant seamounts were missed or ignored | We agree with the reviewer that the information available is limited and that there is a possibility that some significant seamounts were ignored. Due to the resolution of the global bathymetric information available [30”; 3] it is not possible to detect the absolute number of shallow seamounts in the region (Peñaherrera-Palma et al. 2018). For this reason, we combined different information sources that allowed us to be more certain about the identification and location of many of the shallowest seamounts along Cocos Ridge: 1) Seamount catalog (<https://earthref.org/SC/>); 2) National Center for Environmental Information of the National Oceanic and Atmospheric Administration (<https://www.ngdc.noaa.gov/mgg/bathymetry/maps/nos_intro.html>); 3) Depth sounder of the research vessel during the expedition (up to 600 m); 4) the captain’s marine nautical chart and 5) off-line electronic navigation charts from Navionics app. In any case, we also added the following sentence at line 531: *“**Extensive bathymetric research is necessary to identify biologically significant seamounts along Cocos Ridge”* |
| Table 1 | 151 | massive range variability in drift seems an important factor affecting the performance of baited systems, is it considered? | Based on the reviewer’s comments we have added generalized linear models where drift speed is added as a predictor for LPS richness and abundance |
| 159 | 173 | It’s not clear how this reference is useful in this context | We agree with the reviewer. This was a mistake. We intended to add this other reference: Bouchet PJ, Meeuwig JJ. Drifting baited stereo-videography: A novel sampling tool for surveying pelagic wildlife in offshore marine reserves. Ecosphere. 2015;6(8). The reference has been changed. |
| 172 | 189 | !not independent of each other.... each bait could influence the remaining, especially the up stream ones. | We agree with the reviewer. For this reason, we considered each long-line with 5 pelagic BRUVS each as an independent replicate (i.e. we extracted the maximum MaxN of each species among the 5 BRUVS of each long-line). This is explained in the methodology (video and data analysis) |
| 172 | 189 | is this the distance between each BRUV within one long line setup? what was the distance between the two long lines? | See previous comment. The distance between two longlines was 1.6 ± 1.04 km (mean ± SD) (see line 204) |
| 176 | 193 | in table 1 N is the number of deployments ranging between 9 and 20. here it is stated that maximum of 4 deployments, equivalent to 20 BRUVS were made per site. Please clarify | We agree with the reviewer and fixed the error at Table 1 where we defined N as the number of BRUVS (not deployments). |
| 176 | 193 | deployments always started in the same coordinates? drift in the same direction? there is only one set of coordinates per site with N deployments. | The coordinates of each deployment and the drifting direction was different at each site. The set of coordinates per site at Table 1 indicates the location of each seamount. The initial coordinates of each deployment was not originally presented in the manuscript. However, we added the required information in S1 Table at Suplementary Materials. At S1 Table we indicate the initial coordinates of each deployment but no drifting direction since we didn’t measure this parameter as a predictor variable. |
| 177 | 195 | however, in table 1, Las Gamelas2 has N=9.  Also in table 1, only 3 sites have N=20.  Please address the inconsistency between text and table1. | We added the following sentence to clarify the inconsistency noted by the reviewer: *“Due to equipment losses or video failures, the data from few BRUVS units were not included in the analysis. For this reason, less than 20 BRUVS are presented at Table 1 for Paramount, Medina 1, Medina 2 and Medina 3 although 4 deployments were conducted at each site”.* |
| 178 | 199 | This is confusing. A total soak time of 11.7h among 9 BRUVS equates to 1.8h per BRUV on average (assuming that N=9 BRUVs and not long lines), but 55.1h per 20 BRUVS equates to 2.75h deployments, on average, which is longer than the camera battery autonomy. Please clarify | We used battery backpacks for each GoPro that extend the battery life up to 3 hours approximately. We have changed the sentence to *“to extend its recording time between 2 and 3 hours”* (line 167)*.*  Differences on soak times between each BRUVS were associated to logistical reasons more than the survey design. To account for the differences of soak times among BRUVS and seamounts, we divided soaktime / MaxN and use MaxN hr-1 in all the analysis. |
| 180 | 201 | fig 2 only represents the shallow 10m set up | Yes, we are aware of that. We consider it is no necessary to present the deep set up at 25 m since it is exactly the same as the shallow one. The only thing that changes is the depth level. We added this sentence at Fig 2. To clarify: *“The deep deployment is the same as the shallow one but BRUVS units are at 25m depth.* |
| 181 | 201 | not sure what this means, please develop further. | We corrected the sentence to clarify: *“All deployments entered the water upstream of each seamount to ensure that the sound of the vessel engine was not present when the units drifted over the sampling sites”.* |
| 182 | 204 | among 2 long lines? | Yes. See reply to comment at line 172 |
| 191 | 213 | how variable were the drifting speeds? | The average of the drifting speed at each seamount is shown at Table 1. However, we added the drifting speed of each deployment at Supplementary Materials (S1 Table). |
| 192 | 214 | what was the area (size) considered to average the ChlA concentration. Often, the most productive areas associated with localized upwelling or seamount effect are not associated with the seamount summit, thus its important to make sure the area measured contains/reflects the relative proclivity differences among seamounts | The area was 4 km around each deployment. The sentence was corrected in order to clarify: “*Mean concentrations of chlorophyll-a around each deployment (spatial resolution of 4.64km) was extracted from NASA’s Goddard Space Flight Center and the Ocean Biology Processing Group using the ERDDAPP open source data server* (Table 1; S1 Table). Data from ERDDAPP was extracted using the *rxtracto* function from the *rerdappXtracto* package” |
| 212 | 236 | It is a bit repetitive since this point was made clear in the introduction | We agree and have deleted the sentence |
| 215 | 237 | While this approach is widely used it assumes that MaxN is linearly correlated with soak time, which in arguably not the case as the typical function is a saturation one, ie, after a given soak time MaxN is constant. Thus, for any case when the soak time is longer than the point of inflection for MaxN, the standardized MaxN/h will be underestimated. It would be interesting to see some comments or further justification as to why the authors believe this is not the case, for example by showing the longer soak times are below the inflection point or some other means of statistical analysis, perhaps by comparing truncates soak times with resulting MaxN and the total soak time MaxN | Using MaxN hr-1 is comparable to using catch per unit effort (CPUE) when analyzing data from fishery-dependent survey methods, which has been widely used in literature. MaxN hr-1 has been widely used in many studies using BRUVS in order to standardize the survey effort between sites, seasons, locations, etc…  For example:  Sherman, C. S., Heupel, M. R., Johnson, M., Kaimuddin, M., Qamar, L. S., Chin, A., & Simpfendorfer, C. A. (2020). Repeatability of baited remote underwater video station (BRUVS) results within and between seasons. *PloS one*, *15*(12), e0244154.  Ruppert, J. L., Travers, M. J., Smith, L. L., Fortin, M. J., & Meekan, M. G. (2013). Caught in the middle: combined impacts of shark removal and coral loss on the fish communities of coral reefs. *PloS one*, *8*(9), e74648.  Espinoza, M., Cappo, M., Heupel, M. R., Tobin, A. J., & Simpfendorfer, C. A. (2014). Quantifying shark distribution patterns and species-habitat associations: implications of marine park zoning. *PloS one*, *9*(9), e106885.  MacNeil, M. A., Chapman, D. D., Heupel, M., Simpfendorfer, C. A., Heithaus, M., Meekan, M., ... & Cinner, J. E. (2020). Global status and conservation potential of reef sharks. *Nature*, *583*(7818), 801-806.  This is a useful metric since it allows you to make meaningful comparisons without having to use a specific soak time (i.e. 70 min) which might not be possible for every replicate (sometimes battery dies before that, or sometimes you retrieve the cameras after that).  However, based on the reviewer’s comment we have add a supplement figure (S1 Fig) where we show a linear relation between MaxN accumulation rate and soak time for all organisms (S1 Fig. A) and only LPS (S1 Fig. B). These graphics show that the MaxN increases continuously with the soaktime without reaching an inflexion point allowing us to use MaxN hr^-1^ without underestimating relative abundance. |
| 219 | 240 | how thus this relates to table 1 where N can be as high as 20, does it mean 20 independent deployments, each containing 5 BRUVs? | We have made the pertinent changes through the manuscript to clarify this point. For example, we have added a new column at Table 1 for deployments in addition to the column N which refers to the amount of BRUVS unit per deployment. So, we obtained 32 independent deployments containing 5 BRUVS each (most of the times, see next comment) |
| 220 | 242 | Please explain better, Its not clear why standardized MaxN would be summed across seamounts if the intention is to compare seamounts. | We have changed “across” for “per” clarifying that standardized MaxN per species obtained per deployment was not summed across all seamounts, but per each seamount where each deployment was launched. The final sentence looks like “The maximum MaxN·hr-^1^ of each species per deployment was summed per seamount and ecological group for comparison purposes”. So for example, the maximum MaxN · hr-1 of all species from deployment 1 from Paramount where summed to obtain a final value of MaxN · hr -1 at that seamount. The same was done per ecological group. |
| 224 | 246 | is this each BRUV or deployment (long line of 5 BRUVS)? | The reviewer is right. We have deleted the word BRUV because we are referring to each deployment (5 connected BRUVS) |
| 226 | 248 | same as previous comment | We have changed “BRUVS” per “deployment” |
| 254 | 300 | meaning that now all deployments resulted in data from 5 BRUVs?  In the methods section this need to be mentioned as the sampling effort has some slight variations. | We have added a new column at Table 1 specifying number of deployments to clarify. We have also clarified why some deployments had less than 20 BRUVS at line 195 |
| 262 | 309 | what was the proportion of threatened LPS? its a relevant metrics as the LPS are the focus of the research | All of the threatened species were LPS. We have added this sentence to clarify: “*Of all LPS detected, 4 species (30%) were threatened based on the International Union for the Conservation of Nature (IUCN) Red List assessments* *All small teleosts detected are classified as either low concern or not evaluated (Table 3)*” |
| 287 | 333 | is this actual individual BRUV or longline set of 5 BRUVs | Since we were referring to longline set of 5 BRUVS, we have changed “N” per “D” to clarify. This is the same nomenclature used in Table 1. |
| 305 | 351 | the curves look like a representation of the raw data. why not represent the "model" with some measure of confidence limits? this would make the observations about the inflection points potentially more robust | We have added confidence limits into both graphics |
| 309 | 355 | always? in that case all the N values in table 1 should be tipples of 5? | The reviewer is right, although all deployments had originally 5 BRUVS, not always we extracted 5 valid video files from each deployment. Some of the videos were damaged and in one occasion we lost an entire unit. We have clarified this at line 195. Therefore, we don’t consider necessary to clarify this again in the figure caption. The idea here is that the reader has a general idea of what is a deployment when reading the figure caption. |
| 339 | *The original sentence was deleted*  *(388)* | Wines SL, Young MA, Zavalas R, Logan JM, Tinkler P, Ierodiaconou D. Accounting for spatial scale and temporal variation in fish-habitat analyses using baited remote underwater video stations (BRUVS). Marine Ecology Progress Series. 2020 Apr 23;640:171-87.  Sherman CS, Heupel MR, Johnson M, Kaimuddin M, Qamar LS, Chin A, Simpfendorfer CA. Repeatability of baited remote underwater video station (BRUVS) results within and between seasons. Plos one. 2020 Dec 17;15(12):e0244154. | The first paper suggested by the author used predictive modelling approaches to understand how marine species and communities respond to environmental heterogeneity. By including Generalized Linear Models in this paper, we expect to have addressed the reviewer’s concern about the analysis methods on this study.  Both suggested papers mention the importance of considering spatial and temporal scales into BRUVS analysis. We have acknowledged the limitations of this study because of the short spatial and temporal scale (see comment at line 137). We have also recommended surveying twice a year for future studies in the region to incorporate seasonal variations. |
| 367 | 413 | Overall, this first section is oversized and fragmented. I feel it should be rewritten considering the aims of the study and more focused on the relevant results.  its important to situate these results in comparison with similar studies to convince the reviewer that despite the sampling design limitations, the results are valuable information. This section should be kept short and objective. | All the Discussion has been rewritten based on the reviewer’s comments |
| 369 | 413 | or any other physical, biological, ecological or environmental drivers for that matter. I do realize the significance and importance of seamounts in structuring the distribution patterns of LPS in the open ocean, I feel the authors fail to recognize that there are other factors that may be equally relevant. | We agree with the reviewer and have changed the sentence to: “ *Identifying critical habitats in open-ocean ecosystems is of paramount importance to counteract marine predator defaunation* [3,6,38,64,65]*”* |
| 370 | 414 | This reference does not represent well this sentence, please replace with a more suitable one | The sentence and references have been changed. See comment above. |
| 374 | 423 | shouldn't this comparison focus only on LPS since it is the focus of the research and consistent with the discussion bellow | We agree with the reviewer and the focus has been changed to LPS. |
| 377 | 422 | in line 254 authors indicate 32 deployments | We have corrected the error and changed to 32 deployments |
| 388 | 431 | please add a reference to support the statement | The reference has been added: Peñaherrera-Palma C, Arauz R, Bessudo S, Bravo-Ormaza E, Chassot O, Chinacalle-Martínez N, et al. Justificación biológica para la creación de la Migravía Coco - Galápagos. Portoviejo, Manabi, Ecuador; 2018 |
| 398 | NA | it is not clear to each MPAs the authors are referring to | The sentence has been removed from the Discussion. |
| 435 | 554 | A standardized BRUV sampling protocol will not take into account the inter annual variability in environmental conditions, low salinity etc. these and other variables of significance should indeed be considered in the sampling design according to the goals of the study. I do agree that this study can be a reference for future studies in the region using similar methodology. | We agree with the reviewer. We have deleted the information relative to the particular environmental conditions of the ETP and only mentioned that this study can be a first reference for future studies using the same technique. |
| 483 | 620 | why have the authors chosen these two depths for he sampling design? I hypotheses that this is a sampling designed standardized for a specific global monitoring program, not ideal for the region. if that is the case, then it should be stated clearly. | We have deleted this sentence from the discussion and added this information at line xxx: “*Prior to standardize a camera depth level for studies using drifting-pelagic BRUVS in the ETP, we recommend surveying above and below the thermocline (25 m) since different assemblages may vary along depth gradients [81]. If is not possible to evaluate different depth levels due to time or resource based restrictions, we recommend suspending the cameras at 10 m depth following the standards recommended in Australian waters, a pioneer region for the use of this technique [36].”* |
| 491 | 613 | this a know driver of reef fish aggregations at reefs, not likely to be the case here. The reference study does report to reef associated fishes | We have corrected the sentence: “ Such variations could be attributed to species-specific behavioral adaptations such as foraging tactics or predator avoidance [53,107,108]. |
| 492 | NA | too vague, difficult to understand the point | We have deleted that sentence |
| 505 | NA | while the authors suggest this study should be a reference or standard for future studies in the region using similar methods, they also recognize that the sampling design used has important limitations, for example regarding sampling depth in respect to ocean stratification. I suggest addressing this conflict. | The sentence was deleted. Along the discussion we have acknowledge the study limitations and we have been more specific about which recommendations do we provide for future studies using this technique (see discussion section starting at line 551) |
| 508 | 477 | This section should be the #2, after the shorter introductory paragraphs about the methods and comparison with similar studies | We have changed the order of the sections according to this comment |
| 546 | 523 | why is this? | We have deleted that paragraph. Instead we have added this sentence expressing a similar idea:*“Another possible explanation is that greater fishing pressure surrounding the boundaries of MPAs in the ETP is negatively affecting LPS populations at closer seamounts [99,100]”* |
| 554 | NA | Highly speculative and not related to the work presented | We have deleted that paragraph |
| 573 | 547 | once more the authors suggest the sampling design limitations may explain some of the results, thus in contradiction to the other claim that this study should be the standard for future studies | The actual sentence is: *“Future studies with higher temporal and spatial survey efforts, including seasonal variation [106] and the interannual ENSO variability [50], are necessary to better understand the environmental variations at each seamount and its effect on LPS”*  The recommendations derived from this study are related to sampling design standards such as camera depth level, number of cameras per deployment, distance between replicates, soak time, etc… The number of environmental drivers measured will depend on the objective of each study. At line 545 we do recommend measuring different environmental drivers such as temperature, chlorophyll A, currents (or measure deployment drifting speed or distance), pH and dissolved oxygen. |
| 578 | 627 | This section summarizes very accurately the main findings and limitations of the study. However the same is not true for the discussion section, which is fragmented, long and confusing and does not objectively discuss the results and limitations and exceeds the scope of the study | We appreciate the reviewer’s comment. We hope that this new version is discussing clearly and to the point the main results of this study. |

**Reply to general comment from reviewer 1**

1. My overall impression is that the study is of interest as it present novel and important information for the region that surrounds and connects some of the most important world natural heritage areas and this information has the potential to support conservation and management strategies for a range of iconic and threated species and habitats. Although the sampling design has some limitations, they do not preclude the authors from extracting relevant and useful information.

- Answer: We appreciate the positive comment and agree that the information presented here will be an important reference for future studies towards spatial marine planning in the region

2. However, in my opinion, there are two major issues with the ms as it stands, namely the statistical analysis and the discussion section. The chosen data analysis strategy resulted in a very fragmented analysis which is difficult to follow and makes the overview of the results challenging. I have provided some notes and advise on the ms as believe a modelling approach would have been more adequate.

- Answer: we have considered the reviewer’s comment and have added generalized linear models as part of the analysis.

3. The discussion section is long, fragmented and addresses issues that go beyond the scope of the ms and the data. On the other hand, the conclusions summarize well the results and limitations of the study and is very well written.

- Answer: we have rewritten most of the discussion section. We consider this version is more fluent and straight to the point.

4. For these two main reasons I don’t think this ms in its current form is suitable for publication in PlosOne. Given the relevance and novelty of the study I would recommend a resubmission after major changes.

- Answer: we appreciate the reviewer’s opportunity to resubmit the manuscript and hope he/she found this second version suitable for publication

**REVIEWER 2**

Answers to reviewer’s 2 comments. The location of the comments at the original version (lines 1) and the new version (lines 2) are presented.

| **LINE 1** | **LINE 2** | **REVIEWER’S COMMENTS** | **AUTHOR’S REPLY** |
| --- | --- | --- | --- |
| 28 | 30 | add ‘two’ after these so that it reads ‘of these two oceanic marine …’ | We have added the requested word. |
| 46 | 46; 49 | define ‘large’ and ‘pelagic’ | We have added the requested information. |
| 54 - 55 | 58 | refer to Figure 1 here to help guide the reader | We have added the requested reference. |
| 82 | 87 | The non-expert reader may not understand what is meant by ‘a reduced amount of zeros’. Perhaps replace with ‘a higher probability of recording individuals’, or add in ‘i.e. absences’ after ‘a reduced amount of zeros’ | We agree with the author and added “*(i.e. less absences)”.* |
| 90 | 95 | technically scientists are also stakeholders. Replace with ‘non-expert stakeholders’, ‘citizen scientists’ or similar. | We agree with the author and have added *“non-expert stakeholders”*. |
| 102 | 103 | Capitalize ‘south pacific’ | We couldn’t find “south-pacific” in the sentence, but assumed that the reviewer was indicating to capitalize “Northwestern” which we did. |
| 127 - 129 | 133 | This statement needs to be softened. Many predator species still continue to move into waters deeper than this in this region (as shown by tagging studies). | We agree with the author and changed the statement to *“The ETP is also characterized by a shallow thermocline (often at 25 m) above a permanent barrier of cold hypoxic water which seem to limit the available physical habitat for some predator species”.* |
| 131 | 138 | Where was this period on the ENSO scale (i.e. La Nina, El Nino or neutral)? | We added this sentence to clarify *“when the ETP was transitioning from La Niña (cooler-than-average-waters) to ENSO-neutral conditions (sea surface temperatures near the long-term average)”.* |
| 133 | 142 | It’s slightly confusing to have this statement here, when later on the statistical tests are testing whether abundance and richness differs between shallow and deep seamounts (i.e. why are you testing this if you appear to already know the answer?). Perhaps give a reason here why it is thought that the shallower areas will have increased detection rates (i.e. based on previous studies in other regions), or rephrase. | We agree with the comment and added “ *based on results from [53] and [38]*  *where shallower seamounts (< 400 m) showed significant effects on the aggregation of LPS.* |
| 138 | 145 | Do you know the average error from the NOAA estimates? This would be useful for having an idea of the error associated with the depths of the deeper seamounts. | We agree with the reviewer but the required information is not available at NOAA’s web page: <https://www.ngdc.noaa.gov/mgg/bathymetry/maps/nos_intro.html>  However, at least 5 seamounts were < 600 m depth and therefore were detected with the boat depth sounder. The location and depth of the deepest seamounts were obtained not only from NOAA but from boat navigation charts and from Navionics offline nautical charts (https://www.navionics.com/usa/). |
| 158 – 159 | 171 | ‘after that time is less significant’ sounds a little funny. Maybe rephrase/explain this a little more. | We agree and have rephrased the sentence *“considering the little signs of species accumulation captured in the open ocean using pelagic BRUVS* *after 180 minutes of soaktime [33,37].* |
| 180 - 182 | 203 | Did currents or local subsurface oceanography ever inhibit straight line deployments i.e. did the BRUVs ever drift into each other and/or get entangled together? | We used a sea anchor to stabilize the deployments and avoid entangling among lines. We have added a representation of a sea anchor at Figure 2 and added this sentence into the manuscript *“A sea anchor was used to stabilize deployments and avoid entanglements (Fig. 2)”.* |
| 193-194 | 215 | Were these concentrations downloaded for the day/time of deployment at that seamount? What is the spatial resolution/error of these measurements? | Chlorophyll-a concentrations were extracted per day and per deployment (4.64 km around each deployment). We have added this information to clarify: *“Mean concentrations of chlorophyll-a around each deployment (spatial resolution of 4.64km) was extracted from NASA’s Goddard Space Flight Center and the Ocean Biology Processing Group using the ERDDAPP open source data server”.* Furthermore we have added S1 Table with all the associated information to each deployment. |
| 228 | NA | Specifically state what metric of abundance is being tested here – MaxN/hour? | This sentence has been deleted |
| Not specified | 263 | Was visibility in the water column ever an issue? Did it ever vary between sites, deployment depth, seamount depth or time of day? Worth stating this here as well as in discussion | Visibility was not an issue as it was always clear between 15 and 20 meters for both shallow and deep deployments. We have added this sentence to clarify “*As visibility was always above 15 m in all samples, this parameter was not considered as an influencing factor when making comparisons between day time or camera depth levels*” (line xxx) |
| 256 | 301 | is ‘species’ here referring to all species, or just LPS? Be specific. | We have changed the sentence and the calculations to refer specifically to LPS: “*Our cameras detected LPS on 90.6% of all deployments (N = 32) or 48% of individual BRUVS (N = 132), with the number of species per deployment ranging from 1 to 6 (mean ± SD: 1.8 ± 1.3 species)”.* |
| 270 | 315 | I know they aren’t the focus of this study, but I think it would be better to report these statistics separately for dolphins and sea turtles. | We agree with the reviewer and have reported these statistics separately: *“The least sighted and abundant groups where dolphins occurring at 28% of all deployments (1.8 ± 1.7 ind hr^-1^) and sea turtles occurring at 3.1% of all deployments where only 1 individual was detected (0.4 ind hr^-1^)”.* |
| 280 | 327 | Given only one other individual of a marine mammal species was observed (one black Pacific sea turtle), I’d consider removing these statistics, or at least making it clear that the remaining 3% of the MaxN was due to one turtle sighting. | We have clarified that there was only one turtle sighting (see comment above). Futhermore, we have added this sentence: “The bottlenose dolphin (*Tursiops truncatus*) and the sea turtle (*Chelonia mydas agassizii)* were the only species of dolphins and sea turtles, respectively (Table 3)” |
| 373 | 422 | Consider replacing ‘reduced’ with ‘lower’ | We have changed the sentence to: *“Despite the short term nature of this study”* |
| 378 | NA | replace ‘were’ with ‘where’ | We have deleted this sentence |
| 383 | 427 | slightly confusing so please reword. Perhaps replace ‘are needed’ with ‘being needed’. | We have changed the sentence to: “*Although a more robust sampling design (e.g. high spatial and temporal replication) following previous field studies would allow more meaningful comparisons among regions…”* |
| 391 | NA | replace ‘one of the’ with ‘some of the’ | This sentence has been deleted |
| 394 | NA | switching between using scientific name alone, and common name (scientific name) in this sentence. Use one or the other to remain consistent. | This sentence has been deleted |
| 401 | 378 | very cool! | We appreciate the reviewer’s comment and are satisfied as well with our finding |
| 403 | 454 | Replace ‘even’ with ‘ever’. | We have deleted the word. Now the sentence is: *“For example, our BRUVS captured the first visual evidence of the schooling behavior of S. lewini over two different seamounts…”* |
| 417 |  | replace ‘despite’ with ‘although’ | Changed |
| 422 | 461 | Even between shallow and deeper deployments? | Yes. Plus all the animals got pretty close to the camera allowing us for a good identification most of the times. The only animals that we could not identify was because of their small size and fast movements (small teleosts) but not because of visibility. |
| 467 | 591 | and the mobility or speed of each group. | Added |
| 482 | NA | Again, this is quite a strong statement. Tagging in these regions has not shown limitations of elasmobranchs to these depths here. Consider softening as the casual reader may interpret this as these animals aren’t accessing depths deeper than this. | We have deleted that paragraph. |
| 490 | 614 | Given the relatively low sample sizes and high standard errors, be cautious here, and perhaps state that greater sample sizes/efforts are needed to further understand/validate these trends. Also, replace ‘can be attributed to’ with ‘may be attributed to | We have added this sentence *“However, greater sampling efforts are needed to further validate these trends”* |
| 500 - 507 | 549 | Considering the discussion of the ENSO effect in the method, it would be worth discussing how this may influence species distribution/relative abundance, and discussing the need for annual sampling in order to investigate how this may be driving annual variation. | The sentence has been changed to: *“ However, is more likely that a higher temporal and spatial survey effort, including seasonal variation [104] and the interannual ENSO variability [50], is necessary to better understand the environmental variations at each seamount and its effect on LPS”.* We have also added this information at line 487:*“However, it is important to recognize that our data was taken by the end of La Niña phenomenon where the thermocline depth tend to decrease and the chlorophyll-a levels tend to rise [50] potentially increasing species detection”* |
| 517-519 | 480 | Perhaps add the alternative hypothesis here i.e. that the presence of pelagic assemblages here may be due to this area/ridge representing a migratory corridor between Cocos and the Galapagos Islands. | We have added this information: “*Although we need a higher spatial and temporal replication to elucidate if LPS along Cocos Ridge are positively associated to seamounts presence, our cameras detected LPS (swimming individually or in large schools) in the vicinity of all surveyed seamounts in an open ocean environment where species richness is naturally low [92]. These results favor the hypothesis that seamounts might serve as important stepping stones where LPS aggregate when moving between oceanic islands of the ETP [18,93]”* |
| 527 - 531 | 500 | This sentence is confusing, please reword. | It has been rephrased like this: *“The most common explanation given to this pattern is that shallower abrupt topographies accumulate larger zooplankton biomass over the summit providing sufficient food to maintain higher trophic levels and commercial fisheries [83,94]”* |
| 549 | NA | explicitly state here which seasons have higher fishing efforts | We have deleted this paragraph and now this information is not pertinent. |
| Table 1 | 151 | Include the units for the variables measured in the table headings as well as the caption to facilitate easy reading. | Included |
| Table 2 | 330 | Replace ‘sripped marlin’ with ‘striped marlin’ and capitialise ‘pacific’ in ‘Black pacific turtle’. | Replaced |
| Figure 5 | 978 | What are the errors bars here? Standard deviation or standard errors? | The error bars are standard deviation. It has been added in the figure caption (now S1 Fig 1). |

**Reply to general comment from reviewer 2**

1. This study used drifting pelagic-BRUVs to characterize the distribution and abundance of large pelagic species (LPS) in an offshore seamount system. The manuscript is well-written and the authors do a great job of demonstrating the utility of these systems in surveying highly mobile LPS in pelagic environments while being candid about the limitations of the study.

- Answer: we appreciate the reviewer’s comments

2. As the sample sizes/sampling effort are understandably relatively low, I think some of the conclusions come across as a little strong and some parts are a little too speculative.

- Answer: we have rewritten the discussion and consider we have addressed this issue

3. I have made more detailed recommendations below, and would recommend publication of this manuscript following minor revisions.

- Answer: We have addressed all recommendations and hope the reviewer find this version for publication
