# Supplementary material for: A first assessment of the distribution and abundance of large pelagic species at Cocos Ridge seamounts (Eastern Tropical Pacific) using drifting pelagic baited remote cameras
Source: PLoS One. 2021 Nov 18;16(11):e0244343. doi: 10.1371/journal.pone.0244343 (PMC8601560; doi:10.1371/journal.pone.0244343)
Supplement: Attachment — Submitted filename: Response to Reviewers.docx [file pone.0244343.s013.docx]

**REVIEWER 1**

Answers to reviewer’s 1 comments. The location of the comments at the original version (lines 1) and the new version (lines 2) are presented.

| **Lines 1** | **Lines 2** | **REVIEWER’S COMMENTS** | **AUTHOR’S REPLY** |
| --- | --- | --- | --- |
| 47 | 47 | All marine species technically inhabit the water column. Maybe elaborate slightly more e.g. upper layers of the water column, and/or give a depth range. | The sentence has been changed to “hereafter referred to as species that spend the majority of their lives inhabiting the upper layers of the water column in oceanic waters”. |
| 49 | 51 | As the method for measuring the different species included here varies (e.g. TL versus disc width etc), it may be better to refer to mass here. E.g. Estes et al 2016 referred to marine megafauna as being >45 kg max reported mass, or Andrzejaczek et al 2019 referred to large epipelagic species as >30 kg max reported mass. | We consider this is a completely valid suggestion, however we believe that using body size can also be considered a valid approach for this paper since:  1) Body size has been identified a crucial trait in marine ecosystems that determines ontogenetic diet shifts and the strength of competitive and predatory interactions (Dickie et al. 1987, Pinnegar et al. 2003, Karpouzi & Stergiou 2013);  2) Common length has been identified as a good indicator of the ecological role of marine species (Heupel et al. 2014, Roff et al. 2016);  3) Whereas common length is reported for all species of this paper in Fish Base, body mass is not reported for many species and if reported only the maximum weight is available. We consider using common weight would be more appropriate since the maximum weight could be associated to a single specimen outside the usual weight range for the species;  4) There is only one species that would be measured using disc width (*Pteroplatitrygon violacea*) instead of total length, and therefore we consider the effect of using two measuring methods does not pose a significant effect over the final results of this paper;  5) Ultimately, we consider that using body mass or body size would not change much the classification of the reported marine species into ecological groups since body mass and body size are generally correlated parameters. However, we would consider this recommendation for future studies as we believe is a valid observation.  These are the references:  Dickie, L. M., Kerr, S. R., & Boudreau, P. R. (1987). Size-dependent processes underlying regularities in ecosystem structure. *Ecological Monographs*, *57*(3), 233–250.  Heupel, M. R., Knip, D. M., Simpfendorfer, C. A., & Dulvy, N. K. (2014). Sizing up the ecological role of sharks as predators. *Marine Ecology Progress Series*, *495*, 291–298. https://doi.org/10.3354/meps10597  Karpouzi, V. S., & Stergiou, K. I. (2003). The relationships between mouth size and shape and body length for 18 species of marine fishes and their trophic implications. *Journal of Fish Biology*, *62*(6), 1353–1365. <https://doi.org/10.1046/j.1095-8649.2003.00118.x>  Pinnegar, J. K., Trenkel, V. M., Tidd, A. N., Dawson, W. A., & Du Buit, M. H. (2003). Does diet in Celtic Sea fishes reflect prey availability? *Journal of Fish Biology*, *63*(SUPPL. A), 197–212. <https://doi.org/10.1111/j.1095-8649.2003.00204.x>  Roff, G., Doropoulos, C., Rogers, A., Bozec, Y. M., Krueck, N. C., Aurellado, E., Priest, M., Birrell, C., & Mumby, P. J. (2016). The Ecological Role of Sharks on Coral Reefs. *Trends in Ecology and Evolution*, *31*(5), 395–407. https://doi.org/10.1016/j.tree.2016.02.014 |
| 107 | 110 | Capitalize ‘pacific’ | Done |
| 199 | NA | Be specific, how many BRUVs units were not included in the analysis? | This whole sentence has been deleted in order to simplify the way we presented the experimental design based on the other reviewer’s comment. The current sentence is: “Although our initial goal was to conduct 4 deployments (20 BRUVS units) per seamount, due to logistic limitations, equipment losses or video failures the total survey effort differed by seamount (Table 1)” |
| 322 | 337 | Replace ‘where’ with ‘were’. | Done |
| 398 | 402 | What was the directionality of the relationship with ‘distance to nearest MPA’? Higher or lower richness and abundance with increasing distance? | This information is presented as follows: “Relative abundance of LPS was higher at shallow seamounts versus deep seamounts and increased with increasing distance from the nearest MPA (either Cocos or Galapagos Islands)” |
| 510-514 | NA | Reword. This currently reads that shallower habitats have less habitat suitability than deeper waters in the ETP, therefore suggesting BRUVS deployments should be deeper to capture LPS. | This sentence has been deleted in order to reduce the length of the discussion based on the other reviewer’s comment |
| 517-518 | 462 | Again be specific at the start of the paragraph here about the directionality of this relationship. | The sentence has been changed to “Distance to nearest MPA was also a significant predictor for LPS abundance, which increased at increasing distance from the nearest MPA (Cocos or Galapagos Islands)” |
| 540 | NA | These results aren’t technically the ‘opposite’. Perhaps state that they are ‘inconsistent with [103]’. | This sentence has been deleted in order to reduce the length of the discussion based on the other reviewer’s comment |
| 627 | NA | The thermocline depth will vary with location and time of year. Perhaps state ‘(here 25 m)’ | This sentence has been deleted in order to reduce the length of the discussion based on the other reviewer’s comment |
| 637-638 | 572 | rephrase ‘including a positive association of LPS to seamounts’. This was not explicitly tested here as associations away from seamounts were not also tested here. | This sentence should not be there, it was a mistake. It has been deleted. |

**REVIEWER 2**

Answers to reviewer’s 2 comments. The location of the comments at the original version (lines 1) and the new version (lines 2) are presented.

| **LINE 1** | **LINE 2** | **REVIEWER’S COMMENTS** | **AUTHOR’S REPLY** |
| --- | --- | --- | --- |
| 28-29 | 28-29 | Are you talking about moving between the EEZs or MPAs? Make clearer. | We believe the sentence is already specifying that the movements are between the MPAs as it says “moving between Cocos Island and the Galápagos Islands”. |
| 33-34 | 34 | Just use teleost rather than size specific | We have changed “small teleosts, large teleosts” for “small and large teleosts”. We prefer to differentiate between both groups here as we will continue using this classification through the paper. |
| 125-126 | 133 | Need a source and reference for the cited temperature | Done. The added reference was: “Fiedler PC, Lavín MF. Oceanographic Conditions of the Eastern Tropical Pacific. In: Glynn PW, Manzello DP, Enochs IC, editors. Coral Reefs of the Eastern Tropical Pacific. Dordrecht: 2017. p. 59–83” |
| 189-208 | 182 - 191 | This is quite confusing and hard to follow. Need to consider the ordering and stepwise description of deployments. Quite a lot of averages etc. should these not be in the results. | We have simplified the information provided as follows: “The drifting-pelagic BRUVS units were connected with a superficial tether line resembling a long-line system to maximize survey effort while minimizing the risk of loss and the amount of the tracking devices needed. Two long-lines with 5 pelagic-BRUVS separated 150 meters each were simultaneously deployed several times at each seamount during the expedition (Fig 2). Hereon, we refer to each long-line with 5 connected BRUVS units as one deployment. To assess differences in species detectability between depth levels, simultaneous deployments were suspended at 10 m (shallow) and 25 m (deep) (Fig 2). To assess the effect of time of day on species richness and abundance, deployments were conducted during the morning (6 – 10 am) and the afternoon (1 – 3 pm) at each seamount” |
| 192-193 | 187 | Need to state why you refer to each longline as one deployment. Due to independence of observations? | At this point of the manuscript, we only want to clarify that the term to be used when we refer to 5 connected BRUVS in a longline is “a deployment”. In the next section “Data analysis”, it is specified that each deployment will be considered an independent replicate because the distance of 150m between connected BRUVS units does not guarantee independence. |
| 199-201 | NA | Instead of saying “few” or “less than” state the actual numbers | This whole sentence has been modified. See comment two rows above. |
| 201 | 303 | Should final be total? | Yes. We have made the correction. This sentence has been moved to the Results section. |
| 217-221 | 204 | State the product used, it sounds like MODIS but the details are needed. | The reviewer is right. It was MODIS. We have added this information. |
| 236-239 | 221 | If feels a shame to be removing data here. Could there be other ways to consider this data? If large numbers are seen, this does not impact being included in analysis of species richness. | We agree with the reviewer that total species richness is an ecologically important value. For this reason, the total number of species is presented in the descriptive analysis of this manuscript (S1 Table and Table 2). However, for the statistical models we prefer to only include large pelagic species (LPS) since our interest is to understand which predictor variables are affecting their richness, abundance and distribution. To clarify this, we have added this sentence: “Small teleost richness and abundance data were described, but not included in the statistical comparisons since their higher abundance could mask the distribution and abundance patterns of LPS, which are the group of interest on this study. For statistical purposes, LPS were pooled together” |
| 243-244 | 228 | Can you explain this, how does correlation equal unbiased index? | When using MaxN hr^-1^ (MaxN / soak time) instead of MaxN we are assuming that MaxN is linearly and positively correlated with soak time. If this function reaches a saturation point where MaxN is constant after a given soak time, the standardized MaxN/h would be underestimated. For this reason, we add a supplement figure (S1 Fig) where we show a linear relation between MaxN accumulation rate and soak time for all organisms and only LPS (S1 Fig). These graphics show that the MaxN increases continuously with the soaktime without reaching an inflexion point allowing us to use MaxN hr-1 without underestimating relative abundance.  Using MaxN hr-1 is comparable to using catch per unit effort (CPUE) when analyzing data from fishery-dependent survey methods, which has been widely used in literature. MaxN hr-1 has been widely used in many studies using BRUVS in order to standardize the survey effort between sites, seasons, locations, etc.  For example:  Sherman, C. S., Heupel, M. R., Johnson, M., Kaimuddin, M., Qamar, L. S., Chin, A., & Simpfendorfer, C. A. (2020). Repeatability of baited remote underwater video station (BRUVS) results within and between seasons. *PloS one*, *15*(12), e0244154.  Ruppert, J. L., Travers, M. J., Smith, L. L., Fortin, M. J., & Meekan, M. G. (2013). Caught in the middle: combined impacts of shark removal and coral loss on the fish communities of coral reefs. *PloS one*, *8*(9), e74648.  Espinoza, M., Cappo, M., Heupel, M. R., Tobin, A. J., & Simpfendorfer, C. A. (2014). Quantifying shark distribution patterns and species-habitat associations: implications of marine park zoning. *PloS one*, *9*(9), e106885.  MacNeil, M. A., Chapman, D. D., Heupel, M., Simpfendorfer, C. A., Heithaus, M., Meekan, M., ... & Cinner, J. E. (2020). Global status and conservation potential of reef sharks. *Nature*, *583*(7818), 801-806.  This is a useful metric since it allows you to make meaningful comparisons without having to use a specific soak time (i.e. 70 min) which might not be possible for every replicate (sometimes battery dies before that, or sometimes you retrieve the cameras after that).  To clarify it, we have modified the previous sentence to: “The accumulation rate of MaxN for all organisms and for LPS increased continuously with soaktime without reaching an inflection point (S1 Fig 1), allowing us to use MaxN hr-1 without underestimating relative abundance”. |
| 257-259 | 285 –287  275 - 276 | Why do you use negative binomial for one analysis? Should be stated. In table S2 there is an offset used, which is not stated in the methods – this is a very important piece of information to include. How was this incorporated? In the table it is not stated as being a log of the soak time. IS this correct? The offset should be logged in these model algorithms | We have added this sentence to clarify: “A Negative binomial model was used as an alternative of a Poisson model when overdispersion was detected (variance larger than the mean) (Zuur et al. 2009)”  We agree with the reviewer and have stated the use of the offset in the methods with this sentence: “Since soak time differed per deployment, we used the log transformed values of soaktime (hours) as an offset”. This time we incorporated the offset as the log of the soak time in all models as suggested by the reviewer. We have also clarified this in Table S2. |
| Table 2 | 251 - 269 | This table is confusing – The authors described that certain variables were not included in the modelling approach, however they appear in the table of model variables, and include a symbol which states they were not included. The table should only include the variables tested. | This table was removed and the predictor variables included in the model were described and justified as follows:  “The categorical predictors selected include i) time of deployment (morning 6 – 10 am / afternoon 1 – 3 pm), since diel might affect the vertical movement of pelagic species in offshore habitats [64–67]; ii) camera depth level (shallow 10m / deep 25m), since changes in temperature and dissolved oxygen associated to a shallow thermocline (often at 25 m) in the ETP could be an important predictor of pelagic fishes [52,68]; and iii) seamount type based on summit depth level (shallow < 400m / deep > 400m), since marine predators in other regions have shown a higher tendency to be associated with features shallower than 400 m [38,55]. As visibility was always above 15 m in all samples, this parameter was not considered as an influencing factor when making comparisons between time of deployment or camera depth levels. The continuous predictor variables included in the model were i) drifting distance of each deployment during the soaktime (0.8 – 8.9 km) as a proxy of current intensity, since this parameter could influence the abundance of hammerheads and other shark species [11,14]; ii) minimum distance of each seamount to the boundaries of an MPA (0 – 275.5 km), wether it’s Cocos or Galapagos Islands, since both are centers of marine diversity and abundance of LPS in the ETP [11,20,69,70]; iii) temperature at the camera depth level (22.8 – 29.7 ºC), an important determinant of pelagic fish distribution [71,72]; and iv) median chorophyll-a concentration (0.13 – 0.31 mg m−3), an indicator of primary productivity and available trophic energy [28,71]” |
| 289-292 | NA | This is the first mention of SST, what product was used for this? How were VIF, positive redundancy, and multi-collinearity calculated? What variables was SST correlated with, and why were they chosen over SST? | We decided to not use SST as a predictor variable since information of mean temperature recorded per each BRUVS station (a predictor variable incorporated in the models) is more precise and has a direct effect over the pelagic species observed in the cameras. |
| 293-295 | 263-265 | Are the land points single coordinates? Where are these located, centre of the islands? Could a land shapefile of the islands not be used to get a more accurate measure of distance from land? | We decided to use the minimum distance of each seamount to the boundary of an MPA instead of using the land points as reference assuming that the positive effect of either Cocos or Galapagos Islands extends to its boundary. We used shapefiles of each MPA to get accurate distances between the seamounts and the closest point of each MPA boundary. |
| 297-298 | 279-284 | Differences in AIC were stated as being used, but not fully explained. How were AIC scores used to determine best model? Was there a threshold? | We have added this sentence to clarify: “Models were compared using the Akaike’s Information Criteria (AIC), which measures goodness of fit and model complexity. The lower the AIC, the better the model fits the data [73]. Models with similar AIC values were compared with an analysis of deviance (function anova with a chi-squared distribution). If two models were statistically similar (p>0.05), the most parsimonious model was selected” |
| 308 | 312 | Should 132 be 150? | Yes! The mistake was corrected |
| 315-317 | 319 | What species, what IUCN classification? | We have referred to Table 2 where a list of species with their conservation category is specifically shown.  We specified that the IUCN classification is the one presented by the Red List Assessments. This cite was used: IUCN Red List of Threatened Species [Internet]. [cited 2020 Dec 1]. Available from: <https://www.iucnredlist.org>  After some modifications the final sentence was: “Of all LPS identified to species level, 33% were threatened species (n=4), 58% were non threatened (n=7) and only 9% (n=1) were not evaluated based on the International Union for the Conservation of Nature (IUCN) Red List Assessments [75] (Table 2).” |
| 318-323 | 332-338 | What is the metric ind per hour? This hasn’t been explained. Is this the same as MaxN per hour? | We agree with the reviewer and have changed ind hr^-1^ per Maxn hr^-1^ |
| 394-405 | 398 / 402 | Direction of the relationships need to be described. Variables are being highlighted as having an effect, however the reader doesn’t know which way e.g. distance to nearest MPA is important, but are there more fish nearer or further away? | This information is presented as follows: “Richness of LPS was significantly higher at shallow seamounts relative to deeper ones”. And also: “Relative abundance of LPS was higher at shallow seamounts versus deep seamounts and increased with increasing distance from the nearest MPA (either Cocos or Galapagos Islands)” |
| 458-460 | 528 | Is this really the first video evidence of this behaviour? Or at these seamounts specifically? | Is the first video evidence of hammerhead sharks aggregating in open water ecosystems in the ETP. Previous studies studying sharks at these environments used telemetry or were fishery-dependent. We have modified the sentence to clarify: “For example, our BRUVS captured the first visual evidence of the schooling behavior of *S. lewini* at open water ecosystems of the ETP” |
| 544-545 | 489 | Is drift speed actually included in any model? Track distance is included but not speed from what I can see. | The reviewer is correct. Drift speed is not included in the model. Drift speed has been changed by drift distance. |
| Fig. 1 | NA | EZZ should be EEZ | Done |

**Reply to general comment from reviewer 2**

1. In my opinion, there are some issues that are of concern with regards to publication. The main issue is the statistical analysis. The presentation and description of the methods used is confusing, with important details not described making it difficult to follow the processes

We have described all the statistical methods with more detail and with the appropriate references. By deleting the Wilcoxon Rank sum tests prior to perform the models we believe the presented information is now clearer.

2. The authors use Wilcoxon Rank sum tests to look at individual variable effects on their response variables of either species richness, or relative abundance. Then, depending on the outcome of these tests, some variables were included in a GLM using the same response variables. It is not best practice to multiple hypothesis test, and I don’t understand why relevant variables aren’t included in the model from the outset. If the variable is considered biologically/ecologically/behaviourally relevant, then include it and use the modelling framework to decipher the results.

Initially, we didn’t want to saturate the models with too many categorical variables since our data set is small. However, we have followed the reviewer’s advice of not using non-parametric tests to decide which variables to add in the models. Instead, we added all variables in the models to show their significance. We have also transformed two predictor variables that didn’t have a normal distribution and have add the log of soak time, instead of only soak time as an offset. These changes have resulted into better fitted models presented in this version.

3. Within the supplementary materials table there is an offset listed as being used, but not described. This is an important feature of the model if used, and as it is stated, is not used correctly as an offset should be logged in this framework

We have used the log of soak time following the reviewer’s comment.

4. Also, there are two presentations of the models, one showing AIC values highlighting most parsimonious model (Table S2), and a summary table showing p-values (Table 4) neither of which are fully described as to how the final conclusions were made and why two evaluation methods are being used.

We have better specified how we used the AIC to select the optimal model in the methods. Additionally, we have added this sentence in the results section based on this comment: “Summary results from selected models with the level of significance of each predictor are shown in Table 3 (before was Table 4). The model selection process based on maximum likelihood ratio tests and AIC for Poisson and Negative binomial models is shown in S2 Table”.

5. Two variables were shown to be of importance in the models, but only one is plotted, is there a reason seamount depth was not shown?

Since the best fitted Negative binomial model for LPS abundance had two significant predictors we have combined them in the same graph. We believe this is a better way to show the effect of both predictors together instead of presenting two different graphs.

6. The other main area is the discussion – I feel it is overly long and quite dis-jointed. There is less contextualizing of the main results and more review on the technique as a whole.

We have reduced the discussion quite a bit (approximately 1000 words less) and reorganized the information to be more organized.
