# Supplementary material for: A first assessment of the distribution and abundance of large pelagic species at Cocos Ridge seamounts (Eastern Tropical Pacific) using drifting pelagic baited remote cameras
Source: PLoS One. 2021 Nov 18;16(11):e0244343. doi: 10.1371/journal.pone.0244343 (PMC8601560; doi:10.1371/journal.pone.0244343)
Supplement: Attachment — Submitted filename: Response to reviewers.docx [file pone.0244343.s014.docx]

**EDITOR COMMENTS**

- The main issue belongs to the fact that MaxN hr-1, the response variable is a rate so poisson and negative binomial error structures are no longer appropriate. Based on reviewers’ advices, you may have two main option to tackle this:

1) As you have already accounted for soak time in converting MaxN to MaxN hr-1, you could remove the offset of soak time and fit the model with a more appropriate distribution

2) You could also use the offset of soak time but use the raw MaxN value rather than MaxN hr-1. The raw MaxN value is a true count (integer/discrete) and more appropriate for a Poisson distribution

We have already clarified in the comment 2 of the reviewer 3 that the model was actually performed using MaxN as a response variable and not MaxN hr-1. However, we made the mistake of writing MaxN hr-1 in the text of the manuscript instead of MaxN when describing the models. We have already changed MaxN hr^-1^ by MaxN or simply added MaxN to clarify in the lines 249, 394 and 431.

- One way to determine relative support for one model can be to calculate the differences between its AICc and the smallest AICc (ΔAICc) and scaling these differences into model weights (wAICc). All models with values of ΔAICc ≤ 2 can be presented, since values within this threshold can have similar explanatory power. Another step after this is that those models can actually be averaged together to get a final output. All of these techniques can be implemented in the MuMIn package for example

We have incorporated model averaging following the editor’s comment (see answer to question 4 of reviewer 3).

**REVIEWER 3 COMMENTS**

1. This is the second time I have reviewed this, and the authors have made good efforts to improve the writing of the manuscript, especially the length and focus of the discussion. Unfortunately I don’t feel the authors have addressed my original concerns with regards to the statistical analysis to warrant publication. My comments are focused on the statistical analysis as it is my main concern with the manuscript, and is still very confused and often applying techniques and methods inappropriately.

We have incorporate all suggestions made by the reviewer regarding the statistical analysis and expect to have addressed his/her concerns.

2. A response variable of relative abundance is tested using MaxN hr-1. The authors have created a rate as the response variable and so poisson and negative binomial error structures are no longer appropriate. The best approach would be to model the rate using an offset, however this needs to be used in conjunction with a count response variable – in this case MaxN. The authors have included an offset of logged soak time as an offset, based on comments from the previous version, but with a response variable that is already a rate, therefore not applying this technique correctly, and suggesting not fully understanding the reasoning behind the approach.

We understand the reviewer’s concern and would like to clarify that the model was performed using MaxN as a response variable and not MaxN hr-1. Our error was writing MaxN hr-1 in the text of the manuscript instead of MaxN when describing the models. In fact, in the S2 Table we present all performed models using MaxN as a response variable. We have already changed MaxN hr-1 by MaxN or simply add MaxN to clarify in the lines 249, 394 and 431 of the manuscript.

3. Fixed effects should not be transformed

New models were performed without transforming predictor variables based on the reviewer’s comment and the information in the manuscript describing the transformation of predictor variables was deleted (lines 278 – 282).

4. The authors use the z-statistic from the model summary tables as a measure of significance to remove variables from their model. The values given in the summary tables are not corrected and therefore should not be trusted as a test of significance. This is also a pseudo stepwise model selection, which is a highly criticized approach. The authors then use AIC (should it be AICc due to small sample sizes?), which is a quite different approach and the two are not complementary. This also applies to comparing AIC ranked models with an ANOVA to obtain a p-value to select a model, there are methods more appropriate to AIC ranked model sets.

Model averaging was used instead of the null hypothesis significance testing to compare models and measure the probability of each model to be the best model. All the information regarding the use of the z-statistic and all information from the summary tables has been deleted. Instead we added this information in the methods: *“Model selection was based on the small sample-corrected Akaike’s information criterion (AICc). This approach has been suggested as a useful option for small samples where the ratio of observations to model parameters is low (e.g N/K < 40) [78,79]. Models were ranked based on minimum AICc, detailing changes in AICc with respect to the top ranked model (∆AICc) and model weights (wAICc) [80,81]. Models with values of ΔAICc ≤ 2 were presented, since values within this threshold can have similar explanatory power [81,82]. Model weights were computed as a measure of each model’s strength of evidence where the smaller the wAICc, the lower probability the model is true [79]. The cumulative wAICc was used to identify a 95% confidence set of models and to measure the relative importance of each variable [65]. The larger the sum of the weight value (∑wi), the more important the variable is relative to the other variables [65,83]. Residual deviance and GLM diagnostic plots of standard residuals were used to evaluate the goodness of fit of the resulting models and to determine wheatear models assumptions were met [65]”.* In the results section we have changed Table 3 from showing the summary results from the best models to comparing the models with values of ΔAICc ≤ 2. We have discussed each of these models based on their wAICc, the relative importance of its predictor variables and its residual deviance.

5. The authors state they removed the use of Wilcoxon Rank sum tests to look at effects on relative abundance, however there is still a statement of their use for categorical variables (lines 289-292), even after these are included in the GLMs. These variables are being used to test their influence on relative abundance in a model, therefore this creates a multiple comparison problem, and I’m not sure what is to be gained from getting another measurement of effect of these variables

The Wilcoxon Rank sum test was originally used to compare differences in relative abundance of each ecological group between the categorical predictor variables (seamount depth levels, camera depth levels and diel) and not to test their influence on relative abundance. However, we acknowledge that the way the information was presented it was confusing and that these results are not stated as an objective of this paper. Therefore, we have deleted this analysis and the associated figure.

**REVIEWER 4 COMMENTS**

1. I did not see Figure 4 in any of the documents. In the manuscript it goes from Figure 3 (the pictures of individual species) to Figure 5 (depth and MPA comparisons). I also did not see Figure S4 in any of the documents. The Figure versions I received were also quite blurry, although I suspect this may be an issue with uploading it to the system

We agree there were some errors regarding the Figures citation in the manuscript. We have made the following changes to fix the errors: 1) We have included the caption for Figure 1 (line 115) since that was missing; 2) We have changed Fig 1 for Fig 4 in line 377 since the figure we present is the number 4; 3) We have changed S4 Fig for S3 Fig in line 410. Additionally we have added new supplementary figures (S4 and S5 figures) to show results from the new models (see next comment).

2. The authors state the best fitting GLM for LPS richness was one that included only seamount depth. Upon looking at the table though it would seem that, based on AIC values, model 1 was actually the best. I realize that model 2 has a higher residual deviance and that the authors stated that in the case of models with similar AIC values of one another that the most parsimonious model would be deemed the best fit. However, I would just suggest pointing this out in the results section to reiterate it. Another alternative would be to do model averaging, which is a commonly employed approach for models within 2 AIC values of one another. In the case of this paper it would be the top 3 for LPS richness and top 2 for MaxN. Furthermore, given that model 1 for richness also had MPA distance as a factor I think it would be better to make Figure 5 show depth and MPA distance results for species richness similar to what has been done for MaxN.

We have used the alternative suggested by the author and instead of selecting one single best model we used model averaging to compare models and to measure the probability of each model to be the best model. In this new version we have discussed each of the models with values of ΔAICc ≤ 2 based on their wAICc, the relative importance of its predictor variables and its residual deviance. See the answer to comment 4 of the previous reviewer to see how the information has been presented in the new version of the manuscript.

Seamount depth was the only significant variable on LPS richness. However the first ranked model for LPS richness included seamount depth and minimum distance to nearest MPA as predictor variables. Therefore, and following the reviewer’s comment, we have included both variables in figure 5; one graph only showing the effect of seamount depth and a second graph in the same figure showing the effect of both variables.

3. Lines 486-494: The authors state that environmental drivers were not significant predictors of LPS richness or abundance. However, chl a was actually important in model 2 for MaxN, which was within 2 AIC values of model 1. At the very least some discussion to this would seem appropriate

We have made some modifications regarding the discussion of environmental parameters based on new results from model averaging (lines 525 – 549) where we recognize the potential influence of chl-a on LPS abundance since it was included in the third ranked candidate model with Δi values ≤ 2 (line 544).

4. Lines 495 -496. The authors also state that camera depth of time of day had no significant effect on LPS richness or abundance. However, for LPS richness ‘diel’ was actually an important factor in model 3, which was within 2 AIC values. Similar to point #3 above I feel that some discussion of this is warranted

Similar to what we answered in the previous comment, we have also recognized the potential importance of time of day and camera depth level for LPS richness and abundance since they were included in models with Δi values ≤ 2. We also present and discuss the higher relative importance of time of day compared to other non-significant predictor variables (lines 525 – 549).
